# Metastatic prostate cancer in the modern era of PSA screening

**DOI:** 10.1590/S1677-5538.IBJU.2016.0340

**Published:** 2017

**Authors:** Philip A. Fontenot, Avinash Nehra, William Parker, Hadley Wyre, Moben Mirza, David A. Duchene, Jeffrey Holzbeierlein, James Brantley Thrasher, Peter Van Veldhuizen, Eugene K. Lee

**Affiliations:** 1 Department of Urology, University of Kansas Medical Center, Kansas City, Kansas, USA;; 2 Division of Hematology/Oncology, University of Kansas Medical Center, Kansas City, Kansas, USA

**Keywords:** Prostate-Specific Antigen, Neoplasms, Prostate

## Abstract

**Introduction:**

To characterize initial presentation and PSA screening status in a contemporary cohort of men treated for metastatic prostate cancer at our institution.

**Materials and methods:**

We reviewed records of 160 men treated for metastatic prostate cancer between 2008-2014 and assessed initial presentation, categorizing patients into four groups. Groups 1 and 2 presented with localized disease and received treatment. These men suffered biochemical recurrence late (>1 year) or earlier (<1 year), respectively, and developed metastases. Groups 3 and 4 had asymptomatic and symptomatic metastases at the outset of their diagnosis. Patients with a first PSA at age 55 or younger were considered to have guideline-directed screening.

**Results:**

Complete records were available on 157 men for initial presentation and 155 men for PSA screening. Groups 1, 2, 3 and 4 included 27 (17%), 7 (5%), 69 (44%) and 54 (34%) patients, respectively. Twenty (13%) patients received guideline-directed PSA screening, 5/155 (3%) patients presented with metastases prior to age 55 with their first PSA, and 130/155 (84%) had their first PSA after age 55, of which 122/130 (94%) had metastasis at the time of diagnosis.

**Conclusion:**

Despite widespread screening, most men treated for metastatic prostate cancer at our institution presented with metastases rather than progressed after definitive treatment. Furthermore, 25 (16%) patients received guideline-directed PSA screening at or before age 55. These data highlight that, despite mass screening efforts, patients treated for incurable disease at our institution may not have been a result of a failed screening test, but a failure to be screened.

## INTRODUCTION

Despite being labeled an indolent disease, prostate cancer (CaP) remains the second leading cause of cancer death in American men (1). While most patients with CaP have a favorable prognosis, a minority will have an aggressive phenotype leading to a 2.58% lifetime risk of dying from disease (1, 2). In the United States, patient mortality from CaP has decreased over the past 3 decades (1991-present) based on 2014 SEER data, which is attributed to earlier detection with prostate specific antigen (PSA) screening and improved treatment modalities (1). However, despite widespread PSA screening, there remains no consensus on whether benefits outweigh harms of the test, as data are conflicting. This is highlighted by two large clinical trials that examined the impact of PSA screening on CaP specific survival (3, 4).

The PSA screening controversy has led to various guidelines (5-13). In 2009, the American Urologic Association (AUA) recommended a first PSA test at age 40 for men with more than a 10-year life expectancy and did not specify a screening interval (5). In 2010, the National Comprehensive Cancer Network (NCCN) recommended an initial PSA test at age 40 with a screening interval of every 5 years in low risk men and annually in high-risk men (6). The American Cancer Society recommended an initial PSA between age 40-50 depending on patient risk of developing CaP (7). In 2012, the United States Preventative Services Task Force (USPSTF) recommended against PSA-based screening for CaP, stating that current evidence shows that the harms of PSA screening outweigh benefits (8).

Our study objective was to characterize and describe a cohort of patients treated for incurable disease during the contemporary era of PSA screening at our institution. We sought to determine their initial presentation (localized versus metastatic) as well as their PSA screening status prior to initial diagnosis (guideline-directed versus non-guideline directed).

## MATERIALS AND METHODS

After institutional review board (IRB) approval was obtained (KUMC Study 0000852), we used the tumor registry at the University of Kansas Medical Center (KUMC) to identify all patients treated for stage IV CaP between June 2008 to December 2014. These dates correspond with the initiation of electronic medical records (EMR) at KUMC and represent a contemporary era of CaP therapy. We reviewed patient charts for accurate coding of stage IV CaP, past medical history, demographics, family history of CaP and ECOG performance status. Charts were examined to determine initial presentation with prostate cancer (localized versus metastatic) and their PSA screening status at or prior to diagnosis. For patients with missing data, outside records were thoroughly reviewed. Furthermore, we performed telephone interviews with patients or families for those with insufficient information. Patients with missing data were excluded from analysis.

We categorized subjects into one of four groups based on initial presentation with CaP. Patients in groups 1 and 2 presented with presumed localized disease, were treated with surgery or radiation, and had a late biochemical recurrence (BCR) (>1 year; Group-1) or early (<1 year; Group-2). These men eventually developed metastatic disease. Patients in groups 3 and 4 presented with asymptomatic and symptomatic metastatic disease, respectively, from the onset of diagnosis with prostate cancer.

Next, we evaluated if patients received guideline-directed PSA screening using a cut-point of age 55. During the time-frame of our study, the AUA guideline for PSA screening was a first PSA test at age 40 for men with more than a 10-year life expectancy, the NCCN recommended an initial PSA test at age 40, and the ACS recommended an initial PSA between age 40-50 depending on an individual’s risk of CaP (5-7). We chose a cut-off of 55, as this encompassed these screening guidelines, as well as the most recent AUA guidelines (9). We classified patients into groups A through C based on PSA screening status. Patients in group A underwent PSA screening at or before age 55. Group B included those who presented with symptomatic metastases at or prior to age 55 with no prior PSA test. Patients in group C had their first PSA after age 55 and were considered to not have undergone guideline-directed PSA screening.

SPSS 22.2 (IBM, Armonk, New York) was used to perform statistical analysis, with all p-values reported for 2-sided analysis. Descriptive statistics were utilized to present our data, with chi-square and T-tests to assess significance. Kaplan-Meier curve was created to demonstrate overall survival.

## RESULTS

We identified 252 patients coded as having both prostate cancer and stage IV cancer. 173 men had a diagnosis of metastatic CaP, while 79 had localized CaP plus metastatic disease of another etiology (i.e. bladder cancer) and thus were excluded. Thirteen patients were excluded for incomplete records. No patients that required telephone calls were included in the analysis to decrease the risk of recall bias. We analyzed a total of 160 patients in our cohort (157 with complete records on initial presentation and 155 for PSA screening).

**Figure 1:**
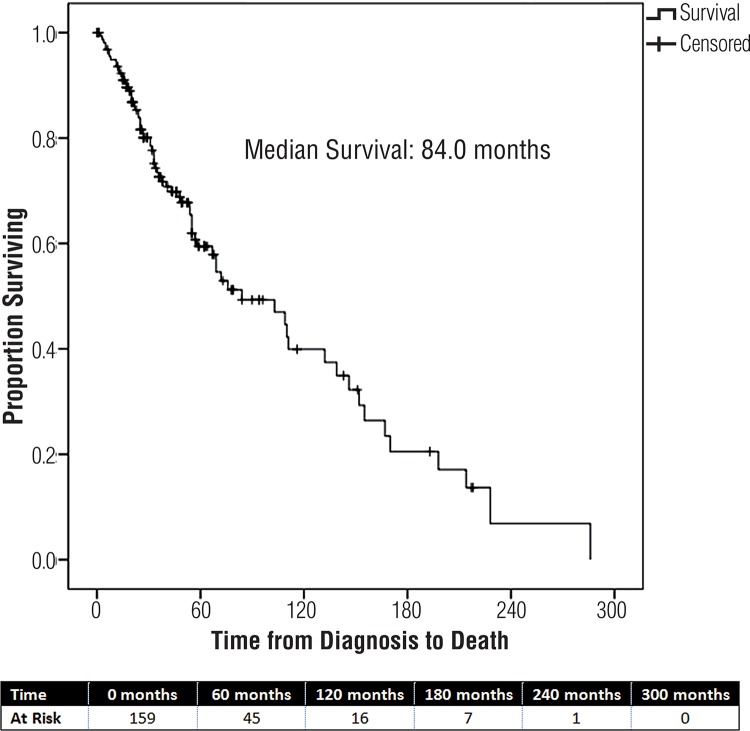
Survival from time of diagnosis.

The mean ages at initial diagnosis of CaP, metastases, and death were 66, 68 and 73, respectively. Most patients were Caucasian (84%) while 12% of our cohort identified as African American. Mean PSA at diagnosis with metastatic disease was 427ng/mL (median 42ng/mL). Mean ECOG performance status was 0.5 (Table-1). At a median follow-up of 38 months (range 0-286 months), 71/160 (44%) of patients died of their disease, with a median overall survival in this population of 84.0 (range: 2-286) months (Figure-1). Patients had a median time from development or diagnosis of metastatic disease to death of 25.5 (range: 0-139) months.

**Table 1:** Patient Demographics.

Patients (n)	160
	
Age at Diagnosis (Mean)	66
Age at Metastasis (Mean)	68
Age at Death (Mean)	73
	
**Race**	N (%)
White	134 (84)
African American	19 (12)
Hispanic/Latino	2 (1)
Asian	1 (0.5)
Native American	1 (0.5)
	
**Family History**	N (%)
Yes	41 (25)
No	109 (68)
	
Mean PSA at Metastasis (ng/mL)	427
Median PSA at Metastasis (ng/mL)	42
	
ECOG at Presentation	Mean: 0.5 (0)

When sorted by initial presentation, 27/157 (17%) patients initially received definitive therapy (radical prostatectomy or radiation) for presumed localized disease, had a BCR greater than 1 year after therapy and subsequently developed metastases. Seven (5%) men underwent definitive therapy for presumed localized disease, but had a BCR lower than 1 year after treatment and subsequently developed metastases. Sixty-nine (44%) patients presented initially with asymptomatic metastases while fifty-four (34%) patients presented to the physician with symptoms of metastatic disease, having CaP confirmed by biopsy (Table-2) In total, 78% of patients in our cohort (Groups 3 and 4) presented with metastatic disease at the outset of their prostate cancer diagnosis.

**Table 2:** Presentation Category.

Group	Number of Patients (%)
1	27 (17)
2	7 (5)
3	69 (44)
4	54 (34)

Groups 1 and 2) presented with presumed localized disease and were treated with either surgery or radiation but recurred late (>1 year; Group 1) or early (≤1 year; Group 2).

Groups 3 and 4) were patients who presented with asymptomatic (Group 3) and symptomatic (Group 4) metastatic disease.

Next, we examined PSA screening characteristics in our patient cohort. Men in Group-A underwent guideline-directed PSA screening at or prior to age 55 and included 20/155 (13%) men. There were 5/155 (3%) men in Group-B who were diagnosed with metastatic CaP prior to age 55, which coincided with their first PSA test 130/155 (84%) patients were included in group C, of which 8/130 (6%) patients underwent screening prior to initial diagnosis of CaP and 122/130 (94%) had their first PSA test at the time of diagnosis with metastatic disease Table-3.

**Table 3:** PSA Screening Category.

PSA Screening Category	N	Percent (%)
A	20	13
B	5	3
C	130	84

Group A) had guideline-directed screening, having their first PSA < or equal to age 55.

Group B) did not have a chance to be screened, as they presented with symptoms of metastasis at less than or equal to age 55.

Group C) did not have guideline-directed PSA screening, having their first PSA above age 55.

We characterized patient’s PSA screening in terms of how they originally presented. In men who presented with presumed localized disease (Groups 1 and 2), 18% (6/34) were considered to have undergone guideline-directed PSA screening, compared to 15% (19/123) in patients who presented with metastatic disease (Groups 3 and 4). Conversely, in patients who presented with presumed localized disease (Groups 1 and 2), 76.5% (26/34) did not undergo guideline-directed PSA screening. In men who presented initially with metastatic disease (Group 3 and 4), 82.1% (101/123) did not have guideline-directed PSA screening (p=0.004). As expected, men who presented with presumed localized disease initially had a lower mean PSA at the time of metastatic development (37.6ng/mL) compared to men who presented initially with metastatic disease (538.1ng/mL), p=0.001.

## DISCUSSION

Our report demonstrates that between June 2008 and December 2014, the majority of patients (123/157 (78%)) treated at our institution for metastatic CaP had initially presented with metastases at the outset of their disease. These findings support a recently published report which found 56% of a 113-patient cohort who died of CaP initially presented with metastases (14). Additionally, 130/155 (84%) of our patients did not undergo guideline-directed PSA screening, defined as having a first PSA at age 55 or younger. The PSA screening rate in our patient cohort is somewhat alarming, especially given that previous studies have estimated the overall prevalence of PSA screening in the USA to be 75% in men over age 50 (13). Our data suggests that patients at our institution who developed clinically significant, metastatic CaP and/or die of disease were more likely to present with incurable disease and not have undergone guideline-directed screening.

Guidelines and position statements on PSA screening vary and there remains no universal consensus on whether or not to perform, when to initiate or end, and at what intervals to repeat PSA screening (5-12). Nevertheless, the trend among organizations is to shift away from mass screening to a limited, opportunistic screening approach in the well-informed male (9). Again, as noted above, we chose age 55 as the cutoff for guideline-directed PSA screening in our study because it encompassed the new 2013 AUA CaP screening guideline and also took into account that the majority of organizations in previous years (during the time-frame of our study) recommended an initial PSA prior to this age (5-7, 9-11, 13).

The ERSPC, first published in 2009 and still on-going, represents the largest randomized CaP screening trial to date. The ERSPC trial showed a reduction in CaP specific mortality in a screened group compared to control in men ages 55-69 (3). The PLCO trial included 76.685 men, and randomized patients to a screened or “usual care” control group. No significant difference in CaP specific or overall mortality was found between the groups (4). Critics of the PLCO study argue that patients in the control group were also screened, 25% and 48%, had DREs and PSAs respectively, which may have negated any benefit of screening (15). Thus, while these two studies are the largest, randomized trials on CaP screening to date, any difference between groups is muddied by the lack of a pure, unscreened control group.

Previous studies have demonstrated the number of patients who present with advanced disease in the modern era of PSA screening has decreased. Catalona et al. reported that 70% of CaP found through PSA screening were organ confined, compared with only 51% of cancers in a referred group of non-PSA-screened patients (16). Furthermore, data from the Center for Prostate Disease Research has shown a decrease in patients who initially present with metastatic disease from 19.8% in 1989 to 3.3% in 1998, which corresponds with the start of widespread PSA screening for CaP in the USA (17).

As noted, there is significant variation in the results of PSA screening studies. Theoretically, large, randomized controlled trials are performed to assess the utility of universal screening efforts. However, is it possible that some cohorts of patients are missed not only by community PSA screening efforts, but also clinical trials? Also, does inclusion in clinical trials bias a population to those who seek out medical care, as they must see a physician to enroll? While we did not look at patient perceived barriers to PSA screening in our study, there are other reports that have attempted to answer this question. Two major barriers that prevent men from getting screened are cost and lack of knowledge about CaP. Additional barriers cited include fear of the rectal exam or cancer, lack of time, resistance to seek healthcare when feeling well, and embarrassment (18-20).

As a retrospective study, this report clearly has limitations. Our patient cohort is small (N=160) and represents a single tertiary referral center, possibly leading to a selection bias for more aggressive and advanced cases. For example, patients who undergo routine PSA screening, are diagnosed with localized CaP, have definitive therapy, develop a BCR and minimal metastatic burden may stay at community practices for androgen deprivation therapy. Conversely, patients who present with more aggressive, metastatic disease may be quickly referred to our center. Second, a retrospective study design relies on the accuracy of medical records and/or patient recall. In order to mitigate possible inaccuracy, we utilized multiple information gathering strategies (i.e., utilizing patient’s EMRs, reviewing outside records, and contacting patients or families). As stated, patients where telephone based recall was the only source of information for initial presentation or PSA screening were excluded to limit recall bias.

As a descriptive study, we focus on conveying the message that patients treated at our institution with diffuse disease are presenting with metastasis at the outset of the diagnosis and the majority of patients in this cohort are not being screened. While we used 55 years of age as the cut-off for what we considered “guideline” directed in this particular study, we do not advocate a certain age at which PSA screening should or should not be used based on these results. Furthermore, it is not our conclusion that men in our cohort would have been cured had they undergone a more standard screening protocol. Clearly, randomized controlled trials have been performed and are on-going to further answer these particular questions.

Despite widespread screening efforts during the time of this study, most patients in our cohort presented with metastatic disease and did not undergo guideline directed PSA screening. These data underscore the notion that despite mass screening during a contemporary time frame, patients treated for incurable disease at our institution may not represent a failure of the screening test, but instead a failure to be screened.
